# The Role of HLA-G in Tumor Escape: Manipulating the Phenotype and Function of Immune Cells

**DOI:** 10.3389/fonc.2020.597468

**Published:** 2020-12-23

**Authors:** Lu Liu, Lijun Wang, Lihong Zhao, Chen He, Ganlu Wang

**Affiliations:** ^1^Department of Gastroenterology, Center for Digestive Diseases, People’s Hospital of Baoan District, The 8th People’s Hospital of Shenzhen, Shenzhen, China; ^2^Department of Critical Care Medicine, People’s Hospital of Baoan District, The 8th People’s Hospital of Shenzhen, Shenzhen, China; ^3^Department of Spine Surgery, Shenzhen Second People’s Hospital, First Affiliated Hospital of Shenzhen University, Shenzhen, China; ^4^Department of Ophthalmology, Shenzhen Second People’s Hospital, First Affiliated Hospital of Shenzhen University, Shenzhen, China

**Keywords:** human leukocyte antigen-G, immune cells, phenotype, function, tumor

## Abstract

Human leukocyte antigen-G (HLA-G) is a non-classical major histocompatibility complex class I (MHC I) molecule, and under physiological conditions, its expression is strictly restricted to the maternal–fetal interface and immune-privileged organs where HLA-G is expected to contribute to establishment and maintenance of immune tolerance. However, the expression of HLA-G has been found in various types of tumors, and the level of its expression frequently correlates with high-grade histology and poor prognosis, raising the possibility that it may play a negative role in tumor immunity. ILT2 and ILT4, present on a broad of immune cells, have been identified as the main receptors engaging HLA-G, and their interactions have been found to allow the conversion of effectors like NK cells and T cells to anergic or unresponsive state, activated DCs to tolerogenic state, and to drive the differentiation of T cells toward suppressive phenotype. Therefore, tumors can employ HLA-G to modulate the phenotype and function of immune cells, allowing them to escape immune attack. In this review, we discuss the mechanism underlying HLA-G expression and function, its role played in each step of the tumor-immunity cycle, as well as the potential to target it for therapeutic benefit.

## Introduction

Human leukocyte antigen-G (HLA-G) is a member of the nonclassical major histocompatibility complex (MHC) class I family. Its expression is initially described as restricted to the fetal–maternal interface, where it protects the fetus from the NK cell-mediated lysis (1). Therefore, the presence of HLA-G on cytotrophoblasts is proposed as a mechanism employed by the fetal to establish and maintain maternal–fetal immune tolerance. Subsequently, it was learned that inhibitory receptors, ILT2 and ILT4, were responsible for HLA-G-mediated inhibitory effect on NK cells (2). Numerous studies reveal that ILT2 and ILT 4 are broadly expressed on a wide range of immune cells, such as NK, T, B cells, and DCs, raising the possibility that HLA-G may exert immunosuppressive function on these cells (3). A series of studies provided strong experimental support for this idea, namely, the interaction of HLA-G with these inhibitory receptors can inhibit proliferation and cytotoxicity of T cells, and modulate the activity of DCs, neutrophils, macrophages, and B cells (4–10). In addition, HLA-G is reported to bind CD8 and KIR2DL4, resulting in the apoptosis of activated CD8^+^ T cells and the inhibition of NK-mediated cytotoxicity, respectively (1, 11).

Currently, HLA-G has been receiving increased attention, since it has been detected in numerous pathological conditions, particularly in tumors, and is expected to have a direct implication in the development of these diseases (12, 13). In this review, we will discuss how HLA-G expression is regulated under normal or abnormal conditions, and highlight the role of HLA-G played in tumor escape as well as the potential to target it for therapeutic benefit.

## HLA-G Biology

### Gene and Protein

The HLA-G gene is located within the MHC class I locus on the short arm of chromosome 6 (p21.31), one of the most polymorphic regions in the human genome (14). However, HLA-G shows only minimal variation, with 80 alleles encoding 21 protein variants, far less than that of classical MHC class I genes (The International Immunogenetics Database-IMGT/HLA, database version 3.41.0) (15).

**Figure 1:**
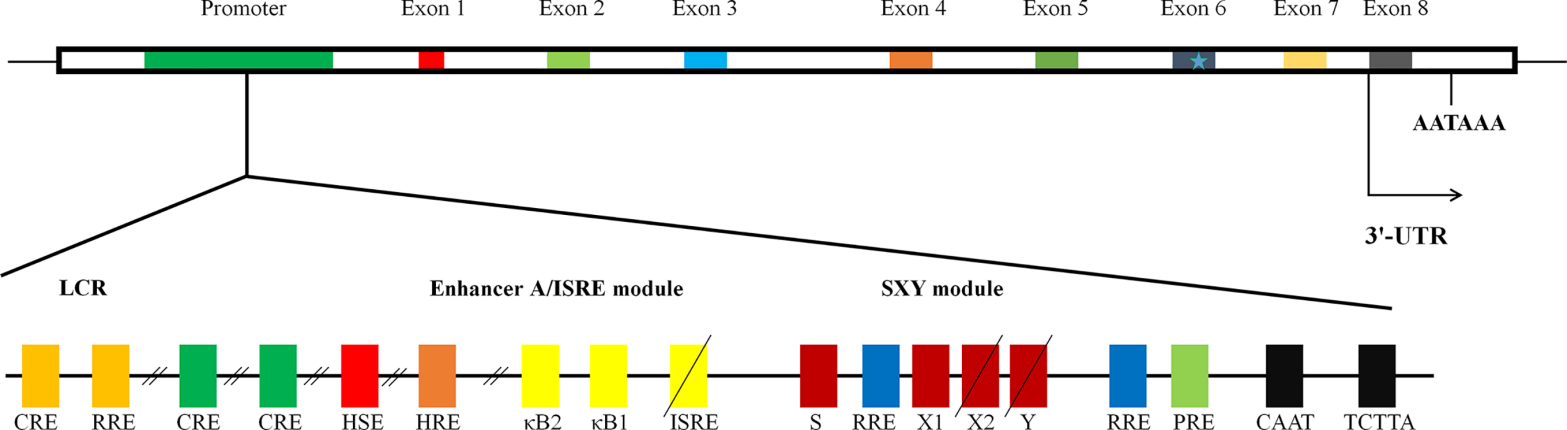
The structure of gene of HLA-G. The schematic diagram of HLA-G gene. It is composed of eight exons and seven introns. Many regulatory elements have been identified and characterized to regulate HLA-G expression. Three conserved cis-regulatory elements, enhancer A, the interferon-stimulated regulatory element (ISRE), and SXY module, mediating the transactivation of classical MHC class I genes; locus control region (LCR), located at least 1.2 kb upstream and crucial for spatio-temporal transcription; heat shock element (HSE), binding heat shock factor 1 in response to heat-shock or arsenate treatments; progesterone response element (PRE), mediating the up-regulation of HLA-G by progesterone during pregnancy; hypoxia response element (HRE), bound by hypoxia-inducible factor in response to hypoxia commonly observed in a majority of malignant tumors; three cAMP response elements (CRE), crucial for basal level of promoter activity of HLA-G; an additional functional ISRE, located at position −746 and capable of transactivating HLA-G following IFN-β treatment; three ras response elements (RREs), served as the binding site for transcriptional repressive factor RREB 1 (ras responsive element binding 1).

The exon/intron organization of HLA-G gene is identical to that of classical MHC class I molecules, which is composed of eight exons and seven introns (14). Because a termination code is located at the second codon of exon 6 of HLA-G gene, most of exon 6 and all of exons 7 and 8 would not be translated into protein (**Figure 1**). HLA-G gene can encode seven isoforms by alternative splicing of primary transcripts, including four membrane-bound (HLA-G1, HLA-G2, HLA-G3, and HLA-G4) and three soluble isoforms (HLA-G5, HLA-G6, and HLA-G7). Soluble HLA-G (sHLA-G) can also be generated through proteolytic cleavage of membrane-bound isoforms. HAL-G1 and HLA-G5 share an identical extracellular structure with classical MHC class I molecules: a heavy chain of three globular domains noncovalently bound to beta-2-microglobulin (β2M) and a peptide (16). The other isoforms do not bind β2M due to lack of one or two extracellular globular domains. It is now recognized that HLA-G spontaneously dimerizes through the unpaired cysteine in its α1 domain (17). Several lines of study reveal that HLA-G dimers bind to ILT receptors with a higher affinity and their inhibitory function is more efficiently than that of monomers (18).

### Expression of HLA-G

Physiologically, the expression of HLA-G is strictly restricted to fetal tissues such as amniotic cells, erythroid precursors, and cytotrophoblasts, and in adults, to immune-privileged organs, including thymus, pancreatic islets, endothelial cell precursors, and erythroblasts (19). HLA-G expression can also be induced during inflammatory-associated diseases such as cancer, transplantation, autoimmune disease, and infection (12, 13).

#### Regulation of HLA-G Expression

Given the important role in immune tolerance, HLA-G expression is tightly regulated at both the transcription and post-transcription levels. Like classical MHC class I genes, HLA-G promoter presents a CCAAT box and an unusual TATA element, TCTTA, controlling basal regulation (**Figure 1**). There are three conserved *cis*-regulatory elements in the MHC class I proximal promoter, namely enhancer A, the interferon-stimulated regulatory element (ISRE), and SXY module, by which transactivation of classical MHC class I genes is finely mediated (20–22). However, HLA-G gene exhibits nucleotide sequence variations, mutations, and/or deletions in those regions, rendering HLA-G unresponsive to NF-κB, interferon regulatory factor 1, and class II transactivator DNA-binding factors (22). Indeed, significant difference in expression distribution and function of HLA-G from those of classical MHC class I molecules suggests the existence of alternative mechanisms beyond the principal MHC class I transactivation mechanisms. To date, several active regulatory elements have been described (21). These include locus control region (LCR), heat shock element (HSE), progesterone response element (PRE), hypoxia response element (HRE), three cAMP response elements, an additional functional ISRE, and three ras response elements, by which the expression of HLA-G is mediated in response to specific signals from environment, by which HLA-G expression is regulated in response to specific environment cues, such as hypoxia and progesterone (**Figure 1**) (21).

Numerous studies have revealed that sequence polymorphism at 5’ upstream regulatory region (5’ URR) and 3’ untranslated region (3’UTR) is also associated with the expression of HLA-G (23–25). In contrast to classical MHC, HLA-G genes is shown to be well conserved in the coding region, but its 5’ URR and 3’UTR display a high level of polymorphism (24, 25). Currently, three variable sites in the 3’UTR, including the 14-base pair insertion/deletion (14-bp INS/DEL), +3142C/G and +3187A/G, have been reported to affect HLA-G expression by modifying mRNA stability or microRNA binding sties as well as the pattern of alternative splicing (26, 27). By analyzing the data from the 1000 Genomes project, 32 polymorphic sites have been identified within 5’URR, of which some—for example, variable site at positions −762 (between a CRE and ISRE)—are close to known regulatory elements and may somehow influence the binding of transcription factors (28). Notably, because polymorphism observed at non-coding regions of HLA-G can affect its expression pattern, it seems to be a genetic factor implicated in the cancer susceptibility. 14-bp INS/DEL, the most extensively studied polymorphism of HLA-G, has been shown to have a strong association with many tumor types, particularly breast and liver cancer, and is generally indicative of an increased risk of cancer (29).

HLA-G gene transcription activity could also be controlled by cis-acting epigenetic mechanisms involving DNA methylation and histone acetylation (30, 31). It has been reported that CpG methylation was associated with the HLA-G silencing in cultured cell lines of various origins and this methylation-mediated repression could be reversed by demethylating treatment for all cell-lines studied (30). Mouillot et al. have demonstrated that the level of histone acetylation in HLA-G promoter of HLA-G-expressing cells is significantly higher than that of HLA-G-deficient cells (31). In addition, several microRNAs, including the miR-152 family (miR-148a, miR-148b, and miR-152) and miR-133, have been characterized to regulate HLA-G expression at a post-translational level (32).

### HLA-G Function

Although HLA-G shows high degree of sequence and structure similarities with classical MHC class I molecules, its main function is not in antigen presentation (18). It is well accepted that the primary function of HLA-G is to serve as an inhibitory ligand for immunocompetent cells, contributing to the establishment and maintenance of tolerance. HLA-G-induced tolerance mainly operates in two mechanisms. The first comprises direct suppression of effector cells: HLA-G directly binds to inhibitory receptors ILT2 and ILT4 that are widely expressed on NK cells, T cells, B cells, and neutrophils in which they mediate negative signaling that counteract immune activation, such as inhibiting T cell proliferation, cytotoxicity, and secretion of cytotoxic mediators (4–10). Moreover, it has been found that HLA-G can induce a shift in the expression of surface proteins present on NK, T, B cells, and antigen-presenting cells (APCs), such that immunosuppressive effects of HLA-G are amplified and maintained. For example, HLA-G significantly up-regulated the expression of its inhibitory receptors, ILT2, ILT3, ILT4, and KIR2DL4 on a broad range of immune cells, and down-regulated the expression of chemokine receptors, CCR2, CXCR3, and CXCR5 on T cells and CXCR4 and CXCR5 on germinal center B cells, which are crucial for the migration, differentiation, and function of immune cells (33–35). A second category of tolerogenic mechanisms comprises the induction of DCs tolerization, which, in turn, renders primed effector cells unresponsive, or promoted the development of regulatory cells. It has been shown that the treatment of human monocyte-derived DCs with HLA-G generated tolerogenic DCs, with a decrease in the level of expression of MHC class II and costimulatory molecules CD80 and CD86 (8). Stimulation T cells with HLA-G-treated DCs could favor to convert naïve CD4^+^ and CD8^+^ T cells to CD4^+^CD25^+^CTLA4^+^ and CD8^+^CD28^-^ regulatory T cells, respectively, rather than effector T cells (8).

In addition, HLA-G can drive macrophages reprogramming to a M2 phenotype, as indicated by up-regulated CD163 and IDO-1, and down-regulated CD86, and these M2-like macrophages suppress T cell responses, such as inhibition of IFN-γ production and proliferation (10, 36). Besides inhibiting effector function of T cells, M2 macrophages are viewed as obligate partners for tumor cell due to secreting a wide range of bioactive mediators, such as VEGF and MMP-9, to promote tumor migration, invasion, and metastasis. Therefore, HLA-G could profoundly alter the phenotype and functional activity of immune cells, and thus may be involved in regulating multiple aspects of the immune response during tumor development.

## HLA-G and Cancer

ctopic expression of “tissue-restricted” HLA-G has been detected in many types of malignancies and is frequently associated with advanced tumor stage and poor prognosis in multiple cohorts of patients with cancer (13, 37). **Table 1** summarizes the studies on the diagnostic and prognostic potential of HLA-G in malignancies (38–74). HLA-G functions as a tolerogenic molecule and contributes to establish an immunosuppressive milieu, enabling tumors to develop without challenge. This may explain why overexpression of HLA-G in tumors correlates with high-grade histology and poor prognosis in patients with breast, lung, ovarian, and pancreatic cancer.

**Table 1:** Clinical studies involving diagnostic and prognostic significance of HLA-G in cancer.

	Diagnostic and prognostic potential	Association with other parameters	
**Breast cancer**	HLA-G expression was significantly correlated with tumor size, nodal status, and clinical disease stage. Patients with positive HLA-G expression had a lower survival rate than those with negative expression.		38
	HLA-G expression was more frequently observed in advanced disease stage and tumor grade.	Increased frequency of Treg was correlated to sHLA-G levels.	39
	For early breast cancer patients with loss of classical HLA class I expression, expression of HLA-G resulted in a worse relapse-free period.		40
**Lung cancer**	HLA-G correlated with high-grade histology.	Loss of classical HLA class I was associated with HLA-G upregulation. IL-10 expression coincided with HLA-G upregulation. NK cells infiltration was associated with loss of HLA class I on tumor cells.	41
	HLA-G expression in non-small cell lung cancer was correlated with lymph nodal metastasis, clinical stages of the disease, and host immune response. Patients with HLA-G positive tumors had a shorter survival time than those with tumors that were HLA-G negative HLA-G exhibited an independent prognostic factor.		42
	Patients with lower level of sHLA-G showed prolonged overall survival.		43
**Esophageal squamous cell carcinoma**	The expression of HLA-G in the tumors was correlated with histologic grade, depth of invasion, nodal status, host immune response, and clinical stage of disease. Patients with positive HLA-G expression had a worse prognosis. HLA-G was an independent prognostic factor.		44
	HLA-G expression was more frequently observed in patients with advanced disease stage. Patients with HLA-G expression had a worse survival. HLA-G could be an independent prognostic factor.		45
**Gastric cancer**	The HLA-G-positive group had a more differentiated histology, less nodal invasion, and earlier clinical stage than the HLA-G-negative group. The 5-year survival rate in the HLA-G-positive group was higher than that in the HLA-G negative group.	HLA-G expression was negatively correlated with NK cells infiltrate.	46
	HLA-G expression in the tumors was correlated with the tumor location, histological grade, depth of invasion, lymph nodal metastasis, clinical stages of the disease, and host immune response. Patients with HLA-G positive tumors had a shorter survival time than those patients with tumors that were HLA-G negative. HLA-G demonstrated an independent prognostic factor.		47
	Patients with HLA-G positive expression had poorer survival at 5 years after operation. HLA-G expression was an independent prognostic factor.	HLA-G expression positively correlated with the presence of tumor-infiltrating Tregs.	48
	Patients with HLA-G-positive primary tumors had a poorer prognosis than patients with HLA-G-negative tumors. HLA-G expression was independent unfavorable factor for patient survival.	Positive correlation between HLA-G expression and the number of tumor infiltrating Tregs and a negative correlation with the number of CD8+T lymphocytes	49
**Colorectal cancer**	HLA-G expression in the tumors was correlated with the depth of invasion, histological grade, host immune response, lymph nodal metastasis, and clinical stages of the disease. Patients with HLA-G positive tumors had a significantly shorter survival time than those patients with tumors that were HLA-G negative. HLA-G demonstrated an independent prognostic factor.		50
**Hepatocellular carcinoma**	Patients with high HLA-G expression possessed shortened survival and significantly accelerated recurrence compared with those with low level in early HCC.	A positive correlation between tumor HLA-G expression and Tregs/CD8+ ratio	51
	Patients with HLA-G-positive tumors had a shorter postoperative survival time than those with HLA-G-negative tumors. HLA-G was an independent prognostic factor.		52
	HLA-G expression in HCC was strongly correlated to advanced disease stage. HLA-G expression was also more frequently observed in elder patients.		53
**Oral squamous cell carcinoma**	HLA-G expression was associated with the clinical tumor stage and lymphatic metastasis. HLA-G level exhibited an inverse correlation with survival rate and a significant direct relationship with clinical stage.		54
**Cervical cancer**	sHLA-G in plasma may have significance in the early detection of cervical malignant lesions.		55
	HLA-G expression was associated with disease stage.	HLA-G expression positive association with human papillomavirus infection and TIL score or the counting of CD57 NK cells	56
	The frequency of HLA-G expression was associated with the disease progression.		57
	HLA-G expression was correlated to the tumor development.		58
**Ovarian cancer**	Patients with HLA-G expression had a worse prognosis. There is a correlation between HLA-G and patient survival and cancer stages.	HLA-G expression was correlated with CA-125 elevation.	59
	A positive HLA-G expression status in tumor tissue is a promising candidate parameter to predict disease recurrence.		60
	HLA-G in effusions was correlated with solid metastases. The reduced expression of HLA-G in post-chemotherapy effusions was correlated with improved survival.		61
	HLA-G was associated with advanced stages and emergence of the first metastases.		62
**Endometrial adenocarcinoma**	HLA-G was associated with advanced stage and metastases.		63
**Bladder cancer**	There was a highly significant increase in the expression of HLA-G on cancer bladder cases with metastatic prostate infiltration.		64
**Melanoma**	HLA-G expression was associated with the disease progression.		65
	There was a correlation of sHLA-G serum level with advanced stages and tumor load. Patients undergoing immunotherapy with IFN-alpha showed an increased serum sHLA-G, whereas other treatment regimens did not influence sHLA-G serum concentrations.		66
**Leukemia**	There was a highly significant relationship between elevated sHLA-G plasma levels and the absence of anterior myelodysplasia and high-level leukocytosis.		67
	Patients with lower level of HLA-G expression had a longer progression-free survival (PFS) time.		68
**Cutaneous lymphomas**	HLA-G expression was associated with high-grade histology and advanced stage.	IL-10 expression was correlated with HLA-G protein presence.	69
**Pancreatic cancer**	HLA-G was associated with both shorter OS and DFS.		70
	There was a positive association between tumoral HLA-G expression and T stage. Diffuse expression of HLA-G in tumor tissues was associated with poor OS.		71
	High level of HLA-G correlated with PDAC aggressive features, such as more advanced stage (TNM Stage II), extrapancreatic infiltration (T3 stage), lymph node involvement, and poor differentiation.	The level of sHLA-G was inversely related to numbers of peripheral activated T cells (CD8+CD28+ T cells).	72
**Glioma**	Tumors with high HLA-G expression were associated with larger tumors and lower mean hyperintensive contrast. Patients with tumors having high HLA-G expression were less likely to have undergone complete resections.		73
**Thyroid cancer**	HLA-G expression was associated with an increased occurrence of lymph node metastasis and capsular invasion. HLA-G could have an independent prognostic value, principally for tumor recurrence.		74

### HLA-G Suppresses T Cell-Mediated Antitumor Immune Responses

**Figure 2:**
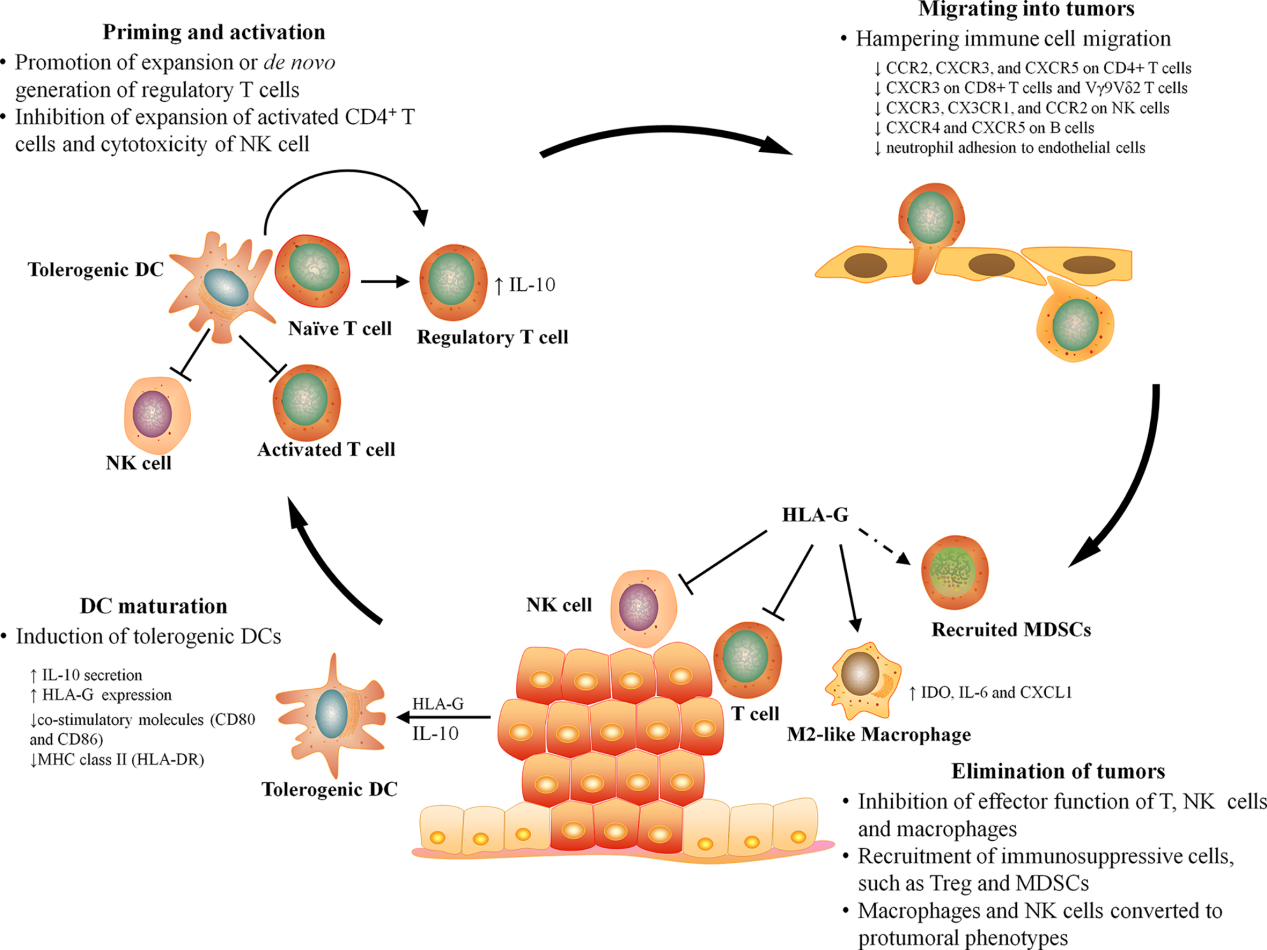
The effect of HLA-G on antitumor immunity. The antitumor immunity can be broadly divided into four major stages, DC maturation, T cell priming, leukocyte infiltration, and elimination. HLA-G can influence every stage of antitumor immune responses by inhibiting DC maturation, inducing T cell tolerance, hampering the migration of leukocyte, and suppressing the effector function and recruiting immunosuppressive cells at tumor sites.

T cells are presumed to be key effectors in cancer immunosurveillance, but to perform this role, they must be properly activated by DCs, home to tumor, and recognize and respond to their target. HLA-G can prevent all of the above requirements for T cell immunosurveillance by directly inhibiting DC maturation, T cell proliferation, and T cell-mediated cytotoxicity, as well as by inducting naïve T cell differentiation into regulatory T cells and recruiting immunosuppressive cells into tumor (**Figure 2**).

#### HLA-G Rendered DCs Tolerogenic

T cell-mediated immunosurveillance is initiated in draining lymph nodes where naïve T lymphocytes differentiate into cytotoxic T lymphocytes upon encountering mature DCs that present tumor-derived antigens with proper co-stimulation signals. A series of studies by Horuzsko group clearly revealed that HLA-G can induce the decrease in the level of expression of MHC class II (HLA-DR) and co-stimulatory molecules (CD80 and CD86) in human monocyte-derived DCs, all of which are required for T cell priming and activation (8, 75). Functional assays further confirmed that these HLA-G treated DCs not only display a reduced capacity to present antigen on MHC II and activate CD4^+^ T cells, but also preferentially promote the differentiation of naïve CD4^+^ T cells into immunosuppressive/regulatory cells (8). Therefore, HLA-G can convert DCs into tolerogenic phenotype. Gregori et al. identified and characterized a novel subset of tolerogenic DCs, termed DC-10, which could also express HLA-G. DC-10 is present *in vivo*, and can be inducible *in vitro* from monocytes in the presence of IL-10 (76). Notably, when cultured in the presence of IL-10, DCs up-regulate the expression of HLA-G and its receptor, and produce more IL-10, resulting in a positive feedback loop.

HLA-G-expressing DCs have been detected in many types of tumor *in vivo*, such as in lung, breast, and ovarian cancer and melanoma, and correlated with a poor clinical outcome (77). *In vitro*, Grange et al. found that CD105^+^ cancer stem cells (CSCs) inhibited the maturation of DCs in a cell contact independent manner when they were cultured together (78). HLA-G released by CSCs was thought to be responsible for this inhibitory effect because the addition of blocking antibodies for HLA-G permitted monocyte-derived DCs to differentiate into mature state. These data, together with experimental studies described above, argue that HLA-G alone or in combination with IL-10 could drive DCs development toward a tolerogenic state at tumor sites.

#### HLA-G Treated or Positive DCs Inducing Tumor Specific T Cell Tolerance

Tolerogenic DCs is viewed as a crucial factor responsible for tumor immune escape. It has been shown that the interaction of naïve T cells with tolerogenic DCs can result in the induction of peripheral T cell tolerance in a T cell-intrinsic (e.g., antigen-specific anergy and deletion) or -extrinsic (e.g., *via* T regulatory cells or cytokines) fashion (79). The effect of HLA-G treated or positive DCs on T cell differentiation, expansion, and effector function has been extensively studied *in vitro* (8, 75, 76). Ristich et al. demonstrated that HLA-G treated DCs changed the cytokine profile (e.g., reducing the production of IL-2 and INF-γ, and increasing IL-10) and inhibited the expansion of activated CD4^+^ T cells and thus rendered T cell anergic (8). LeMaoult et al. also demonstrated that HLA-G-transfected DCs (HLA-G^+^ DCs) not only induced CD4^+^ T cell anergy but also caused the differentiation of CD4^+^ T cells into suppressive cells, as indicated by unresponsiveness to allostimulation in mixed lymphocyte reactions (80). Further phenotypic and functional analysis revealed that these HLA-G^+^ DCs primed T cell were CD4^low^ Foxp3^-^ or CD8^low^ Foxp3^-^, and exerted immunosuppressive function through the actions of IL-10 (81).

#### HLA-G Impacted the Infiltration of Effector Cells Into Tumor

To effectively control the growth of cancer cells, T cells must gain access to, and function within the tumor microenvironment. Migration of lymphocytes into tissues is a complex process that requires highly coordinated interactions, involving chemokine receptors and chemokines, and integrins, selectins, and their respective ligands. A role of HLA-G in regulating chemokine receptor repertoires in T cell was first described by Morandi et al. who demonstrated that HLA-G can significantly down-regulate the expression of CCR2, CXCR3, and CXCR5 on CD4^+^ T cells, and CXCR3 on CD8^+^ T cells and Vγ9Vδ2 T cells (81). Similar result has been found in NK cells, sHLA-G inducing CXCR3, CX3CR1, and CCR2 down-regulation in NK cells (82). Increasing evidence has suggested that these chemokine receptors are crucial for the migration of effector T and NK cells toward inflamed tissues. For example, CXCR3-deficient NK cells fail to migrate toward tumor following activation (83). Additionally, in the germinal centers (GCs) that are the main sites where activated B cells differentiate into memory B cells and plasma cells, CXCR4 and CXCR5 expressions on B cells were significantly down-regulated following exposure to sHLA-G, which limited B cell development by dampening B cell trafficking in GCs (35). It has been reported that HLA-G expressed on endothelial cells inhibited NK cells adhesion and transmigration, two key events which occurred during activated NK cells infiltrating from blood into inflamed sites (84). Mociornita et al. also found that in the model of cardiac allograft vasculopathy, HLA-G is overexpressed in vascular endothelial following everolimus treatment, which leads to the inhibition of HCASMC proliferation and of TNFα-stimulated neutrophil adhesion to endothelial cells at all concentrations (85). However, HLA-G has little effect on the migration of control T cells. Given that a high level of serum sHLA-G was frequently detected in patients with cancer, the importance of HLA-G in hampering the migration of NK and T cells into tumor microenvironment is becoming increasingly appreciated.

#### HLA-G Inhibited T Cell Function at Tumor Site

It has been well-established that HLA-G can directly bind to the inhibitory receptor, ILT-2 and ILT-4, on activated T cells, resulting in dramatically diminishing their proliferative and cytotoxic activities (4–6). Besides direct inhibitory actions, HLA-G can recruit suppressive cells to inflammatory site, by which effector functions of T cells are suppressed. According to the study of Agaugué et al, they developed an HLA-G^+^ xenotumor model by injecting melanoma tumor cells (M8) or M8 transfected with HLA-G into immunocompetent mice, and found that M8 expressing HLA-G grew rapidly, whereas M8 were rejected immediately *in vivo* (86). Analysis of the immune responses following the tumor progression released that HLA-G promoted accumulation and suppressive activity of MDSCs in HLA-G^+^ tumor-bearing mice through engagement of the paired immunoglobulin like receptor-B (PIR-B), the homolog of human ILTs. Therefore, HLA-G can inhibit the antitumor T cell responses in a direct and/or indirect manner.

### HLA-G Converted Innate Immune Cells to Immunosuppressive and Protumoral Phenotypes

Generally, innate immune cells, such as NK cells and macrophages, function as the first line resistance against transformed cells, and activate a much more potent T cell response against tumors. HLA-G is initially characterized as a suppressive regulator for NK cells to induce maternal-fetal tolerance by inhibiting their cytotoxic activity (1). For tumors, ectopic expression of “tissue-restricted” HLA-G is essential for evading NK-mediated killing, since malignant transformation of cells is frequently associated with the loss of expression of MHC class I molecules, rendering them directly susceptible to NK-mediated lysis, as proposed by the “missing self hypothesis” (87). It has been reported that in lung cancer and classical Hodgkin’s lymphoma, a frequent focal or complete loss of HLA class I molecules associated with HLA-G protein expression (41, 88). In addition to exerting direct inhibitory action on NK cells, HLA-G is found to impair NK/DC crosstalk, resulting in a slight inhibition of NK cell cytotoxicity (89). Several studies have revealed an important role of HLA-G in promoting vascular remodeling by activating uterine NK cells that secrete pro-angiogenic factors during early progeny (90, 91). This finding raises the possibility that HLA-G may alter the functional properties of tumor-infiltrating NK cells in a microenvironment similar to that of embryo and thus contribute to the angiogenesis at the tumor sites.

Similarly, HLA-G has long been known to be a potent negative regulator of macrophages (36). Moreover, it has been shown that HLA-G can drive macrophage polarization toward a protumoral phenotype, characterized by increased expression IDO, IL-6, and CXCL1, which could result in the inhibition of T cell response and the tumor progression; that is, HLA-G-polarized macrophages act as tumor-associated macrophages at tumor sites (36, 37). It has been found that in breast cancer and clear cell renal cell carcinoma, macrophages bearing ILT2 and ILT4 receptor, respectively, are present around HLA-G-positive tumor cells, which cooperatively establish an immune-tolerant microenvironment (92, 93). Therefore, HLA-G not only exerts immunosuppressive effects on macrophages and NK cells but also drives reprogramming into tumor-promoting cells.

### The Environmental Factors Activating HLA-G Expression in Tumors

Currently, no clear explanation for the ectopic expression of “tissue-restricted” HLA-G in various types of tumors exists. Because no genetic alternation that is heavily implicated in the upregulation of HLA-G gene, such as copy number gain or translocation, is detected in cancer genome, elevated HLA-G promoter activity is thought to be crucial for its expression in tumors. As described above, there are several *cis*-regulatory elements present in the promoter region of HLA-G gene which may respond to specific signals from the tumor microenvironment (**Figure 1**). For example, HRE can be bound by hypoxia-inducible factor in response to hypoxia commonly observed in a majority of malignant tumors; an additional functional ISR that is located at position −746 is capable of transactivating HLA-G following administration of IFNs for immunotherapy of malignant diseases; PRE has been reported to mediate the up-regulation of HLA-G by progesterone implicated in breast cancer progression; HSE can bind heat shock factor 1 in response to hypoxic stress and induce HLA-G gene transcription. Therefore, tumor microenvironmental factors, such as hypoxia, stress, hormones, and inflammation, have been proposed to activate HLA-G expression in tumor cells. It has been observed that HLA-G expression is gradually downregulated or even lost during long-term culture of primary tumor cells, which provides direct evidence for this hypothesis (41). A series of *in vitro* studies using tumor models have investigated the effect of these microenvironmental factors on HLA-G expression of tumor cells. Mouillot et al. found that hypoxia can activate HLA-G gene transcription in HLA-G-negative cell lines (94). He et al. demonstrated that HLA-G expression in breast cancer MCF-7 cells was upregulated by progesterone but was inhibited by its antagonist (38). Additionally, analysis of HLA-G expression in malignant lesions reveals that upregulation of HLA-G expression frequently correlates with high inflammatory infiltration, which in turn releases a variety of cytokines (91). *In vitro* studies have shown that GM-CSF and IFN-γ secreted by the infiltrating cytotoxic T cells can enhance HLA-G expression in tumor cells. In contrast, by systematically testing the effect of various cytokines in the choriocarcinoma cell line JEG-3, Persson et al. recently found that cytokines studied had no or adverse effect on HLA-G expression (95). These conflicting results suggest that HLA-G modulation by cytokines may be a cell-type specific phenomenon; JEG-3 decreases the production of HLA-G when exposed to the persistence of cytokines with high concentration.

## Conclusion

Many immunosuppressive mechanisms are evolved by the host immune system to protect tissue damage caused by excessive or inappropriate immune activation, but they also provide opportunities for tumor to evade antitumor immune responses. Physiologically, HLA-G expression is strictly restricted to the maternal–fetal interface and immune-privileged organs where it is expected to contribute to establishment and maintenance of immune tolerance. However, overexpression of HLA-G is frequently detected in patients with cancers and thus is viewed as a commonly immunosuppressive strategy employed by tumor to counteract effective immune responses by manipulating the phenotype and function of immune cells, such as DCs, macrophages, and NK and T cells (**Table 2**). As we are now recognizing, HLA-G influences almost every stage of antitumor immune responses, such as T cell priming by DCs, and infiltration and function of effector cells at tumor (**Figure 2**). Therefore, HLA-G may represent an attractive target for therapeutic intervention. Several preclinical studies have provided some evidence that blocking HLA-G/ILTs signaling with antibody or down-regulating HLA-G expression with RNA interference can restore function of immune cells and prevent tumor reoccurrence (37, 96, 97). Currently, a clinical trial in phase I (NCT04485013) is underway targeting HLA-G by TTX-80, a monoclonal antibody, for patients with HLA-G-positive advanced cancers. Dumont et al. recently demonstrated the potential of combination of HLA-G and PD-1/PD-L1 blockade to confer a greater benefit to cancer patients, particularly for those with nonresponsiveness to anti-PD-1/PD-L1 (98). Therefore, combination blocking HLA-G with other immune checkpoints (PD-1/PD-L1 or CTLA-4) represents an inspiring strategy for cancer treatment, which may help overcome the resistance routinely developing in patients treated with a single immunotherapy. Currently, to bring HLA-G blockade therapy into clinical reality, several problems have yet to be solved, such as identifying predictive biomarkers for assessing the therapeutic effectiveness and elucidating the exact ways in which environmental factors and/or genetic changes regulate the expression of HLA-G in tumor or immune cells.

**Table 2:** Significant changes in phenotype and function of immune cells induced by HLA-G.

	Changes in phenotype		Changes in function
	Up-regulated expression	Down-regulated expression	
**NK cell**	ILT2, ILT3, ILT4, KIR2DL4, TNF-α, IFN-γ, MIP-1, MIP-3, IL-1β, IL-6, IL-8, IL-23	CXCR3, CX3CR1, CCR2	Inhibition of cytotoxic function, proliferation, and chemotaxis Induction of apoptosis Increased secretion of pro-angiogenic factors
**CD4+ T cell**	ILT2, ILT3, ILT4, KIR2DL4, IL-3, IL-4, IL-10	CD4, CCR2, CXCR3, CXCR5, TNF-α, IFN-γ	Inhibition of alloreactivity, proliferation, and chemotaxis Induction of anti-inflammatory response by shifting the Th1/Th2 balance Induction of regulatory/suppressive cells
**CD8+ T cell**	ILT2, ILT3, ILT4, KIR2DL4	CXCR3	Inhibition of cytotoxic function, proliferation, and chemotaxis Induction of apoptosis Induction of regulatory cells
**DCs**	ILT2, ILT3, ILT4, KIR2DL4, IL-1β, CD83a	HLA-DR, CD80, CD86, HLA-DM, GILT, CD74, LAMP3, Dynactin 2, Dectin-1, DCSTAMP	Inhibition of maturation Induction of tolerogenic state Induction of T cell tolerance
**Macrophage**	CD163, IDO-1, IL-6, CXCL1, IL-12, TGF-β	CD86	Inhibition of phagocytic function Induction of differentiation toward M2-like phenotype
**B cell**		CXCR4, CXCR5	Inhibition of Ig secretion, proliferation, and chemotaxis
**Neutrophil**		Reactive oxygen species	Inhibition of phagocytic function

## Author Contributions

GW designed work. LL and LW wrote the paper. LZ and CH contributed to critical revision of the manuscript. All authors contributed to the article and approved the submitted version.

## Funding

This study was supported by Shenzhen Foundation of Science and Technology (JCYJ20170307094549868 and JCYJ20180305123929814), Science and Technology Bureau of Baoan (2018JD124), and Funds for Young Scholar of Shenzhen Baoan People’s Hospital (2018A003).

## Conflict of Interest

The authors declare that the research was conducted in the absence of any commercial or financial relationships that could be construed as a potential conflict of interest.
